# Supplementary figures and images for: The INO80 chromatin remodeler sustains metabolic stability by promoting TOR signaling and regulating histone acetylation
Source: PLoS Genet. 2018 Feb 20;14(2):e1007216. doi: 10.1371/journal.pgen.1007216 (PMC5834206; doi:10.1371/journal.pgen.1007216)

S1 Fig

A.

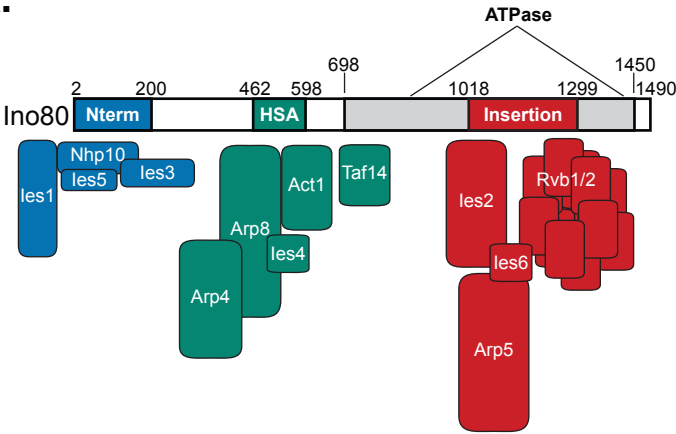

B.

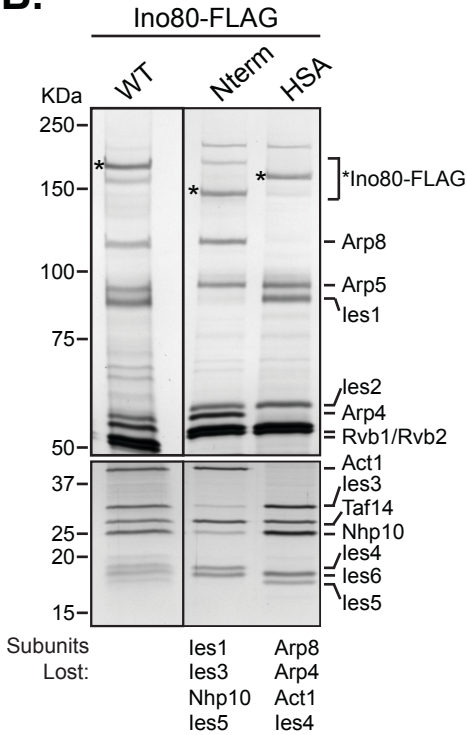

C.

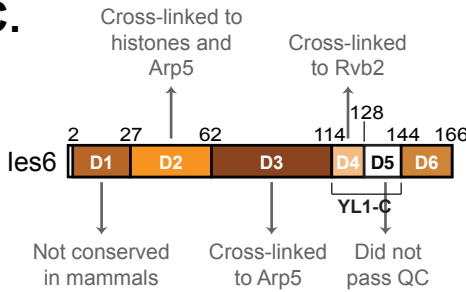

D.

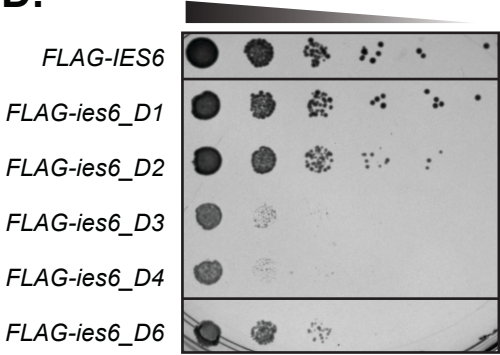

Supplement: S1 Fig — (A) Schematic of the Ino80 ATPase protein domains with subunit binding modules illustrated from previous structural and biochemical studies [22,30]. Ino80 insertion is as described by [103], HSA domain is as identified in [44], N-terminus (Nterm) is amino acids 2–200. (B) Ino80-FLAG purifications from wild-type (WT), N-terminal deletion (Nterm), and HSA deletion strains were electrophoresed on 6% (top) and 15% (bottom) SDS-PAGE gels and identified by asterisk. Proteins were visualized via silver staining. Subunits of the INO80 complex are labeled on the right, molecular mass (KDa) is labeled on the left. Subunits lost from the INO80 complex are identified at the bottom. (C) Schematic of Ies6 gene domains, the YL1-C domain is split into domain 4 (D4) and domain 5 (D5). D5 was omitted from additional assays because EMAP results did not pass quality control (QC). (D) Fitness assay of indicated FLAG-tagged domain mutants described in (C). 1:10 serial dilution of strains were grown for 3 days at 30°C on YPD. (PDF) [file pgen.1007216.s001.pdf]

S2 Fig

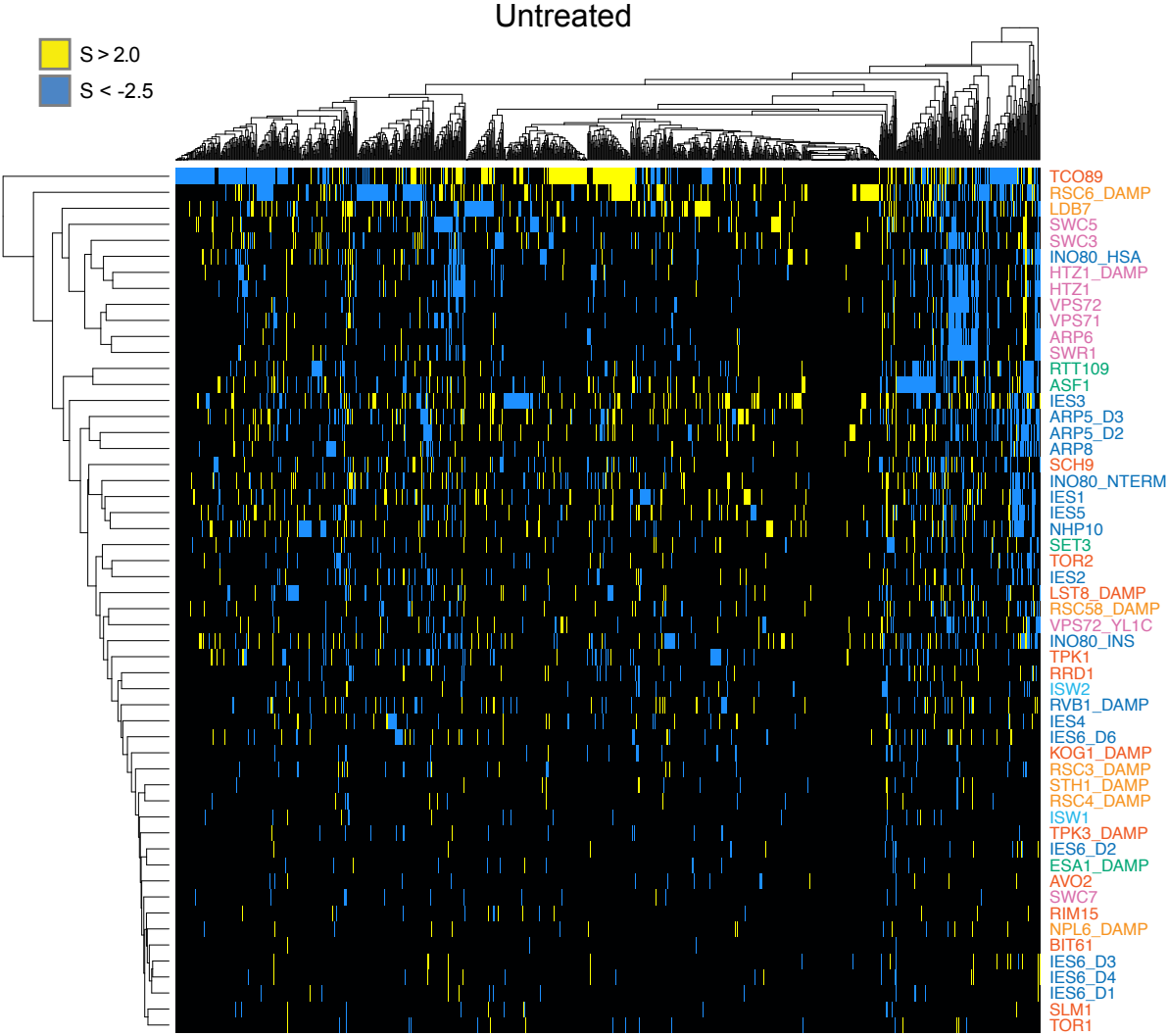

Supplement: S2 Fig — Clustergram of all significant interactions in the untreated static EMAP between the 54 query strains and all test strains with at least one significant interaction. Test strains are along the x-axis. Text colors correspond to the query category annotated in Fig 1B. S1 Table lists all EMAP scores. (PDF) [file pgen.1007216.s002.pdf]

S3 Fig

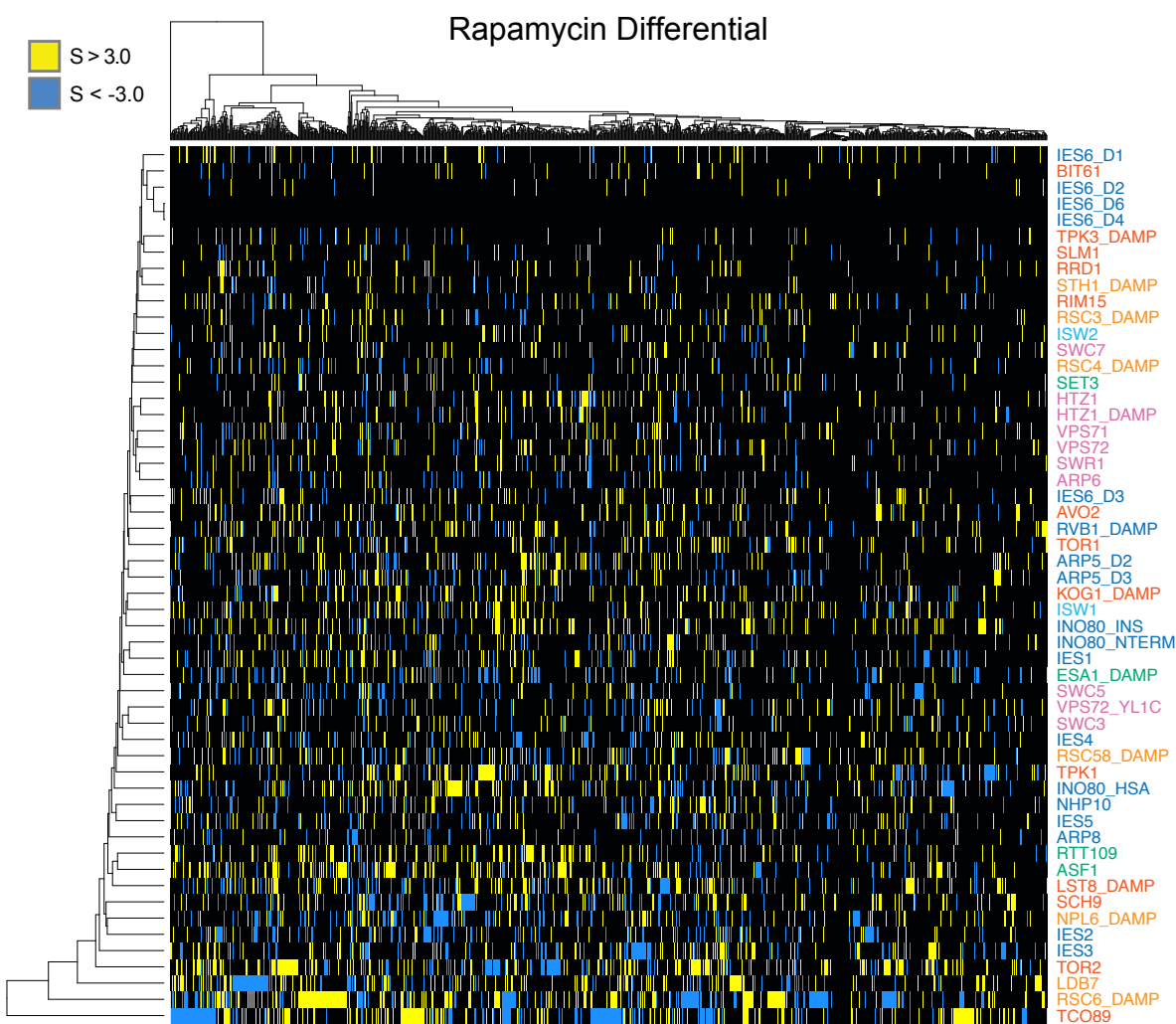

Supplement: S3 Fig — Clustergram of all significant interactions in the rapamycin differential EMAP between the 54 query strains and all test strains with at least one significant interaction. Test strains are along the x-axis. Text colors correspond to the query category annotated in Fig 1B. S1 Table lists all EMAP scores. (PDF) [file pgen.1007216.s003.pdf]

S4 Fig

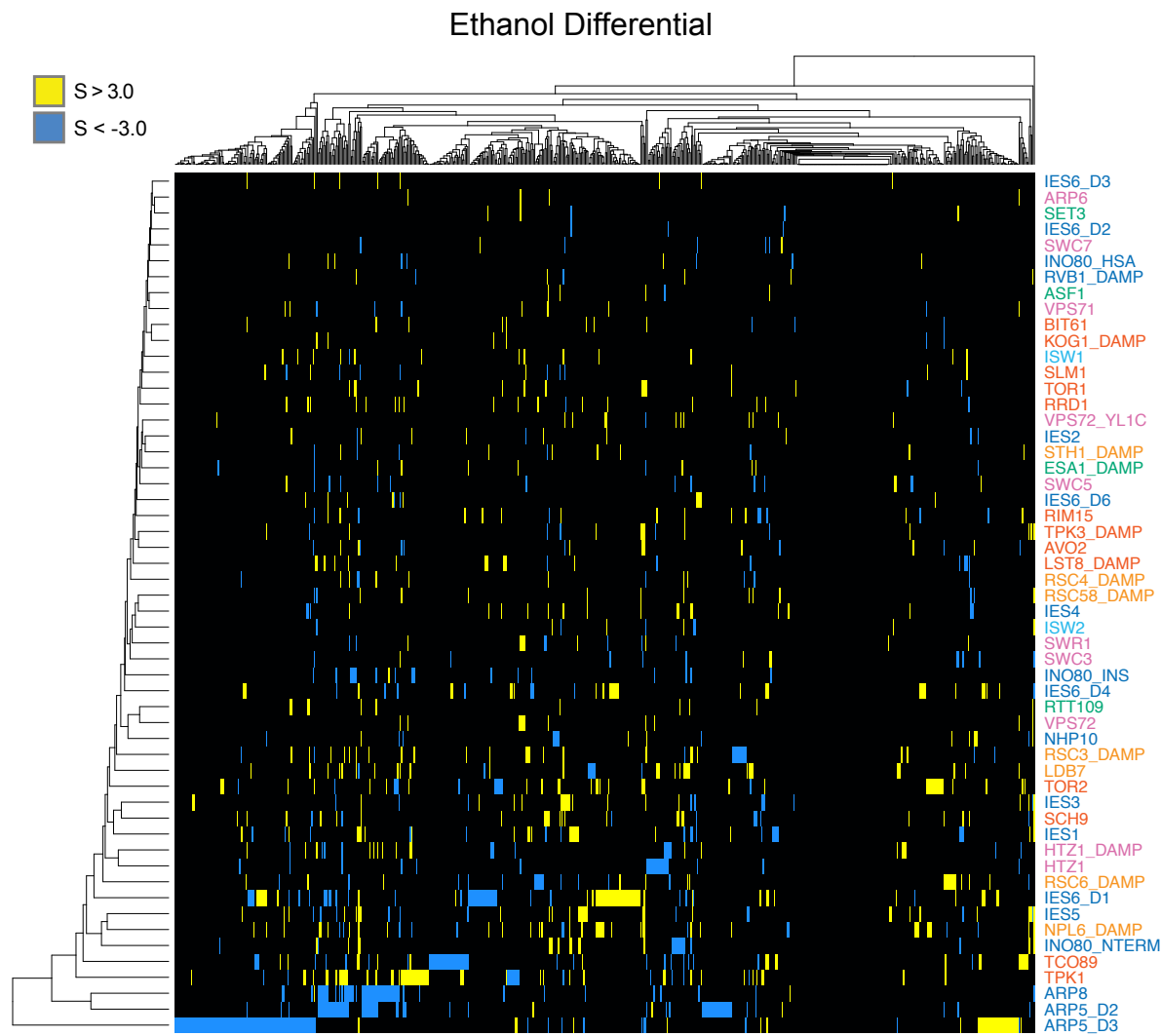

Supplement: S4 Fig — Clustergram of all significant interactions in the ethanol differential EMAP between the 54 query strains and all test strains with at least one significant interaction. Test strains are along the x-axis. Text colors correspond to the query category annotated in Fig 1B. S1 Table lists all EMAP scores. (PDF) [file pgen.1007216.s004.pdf]

**S5 Fig**

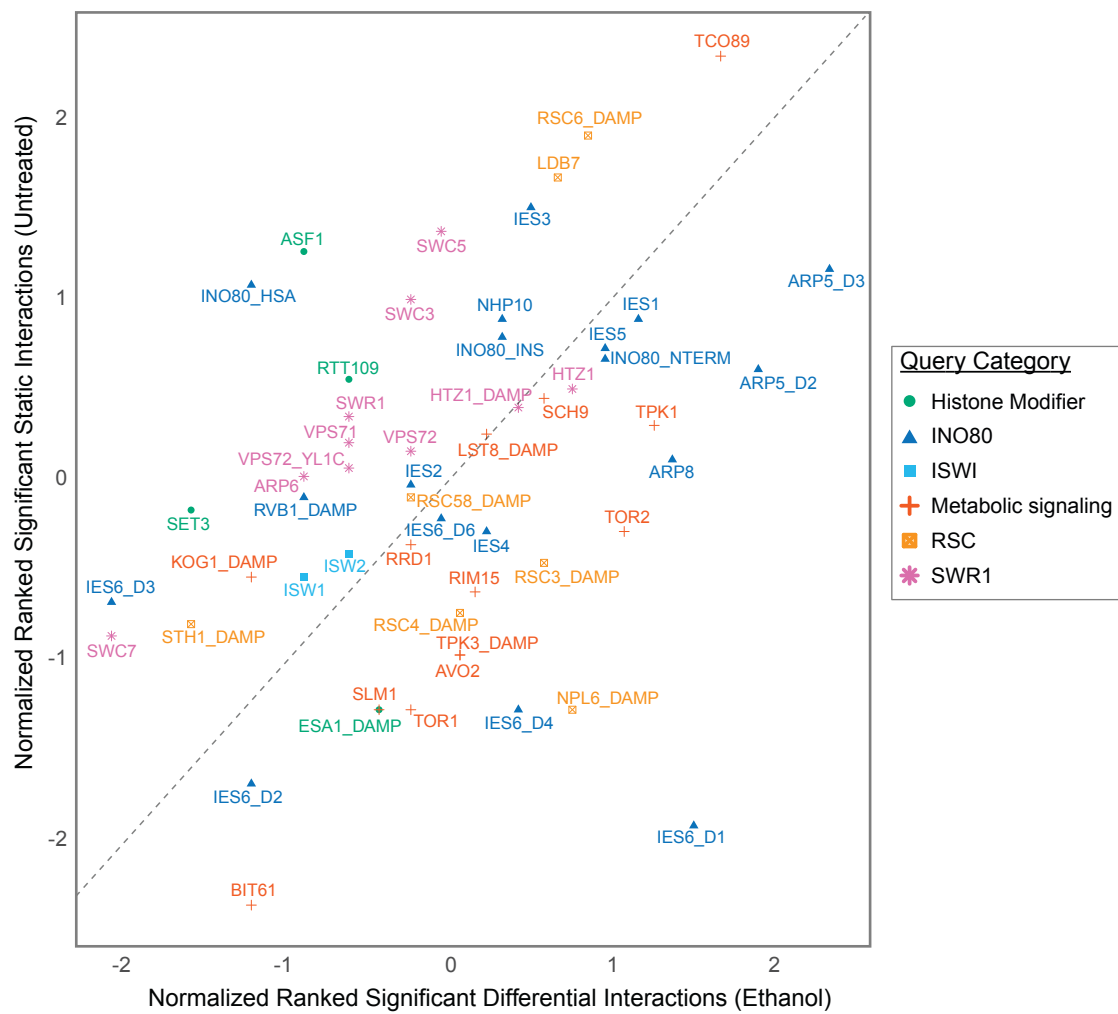

Supplement: S5 Fig — Plot of normalized significant interactions by query gene in the untreated condition and the ethanol differential condition, as in Fig 1F. (PDF) [file pgen.1007216.s005.pdf]

S6 Fig

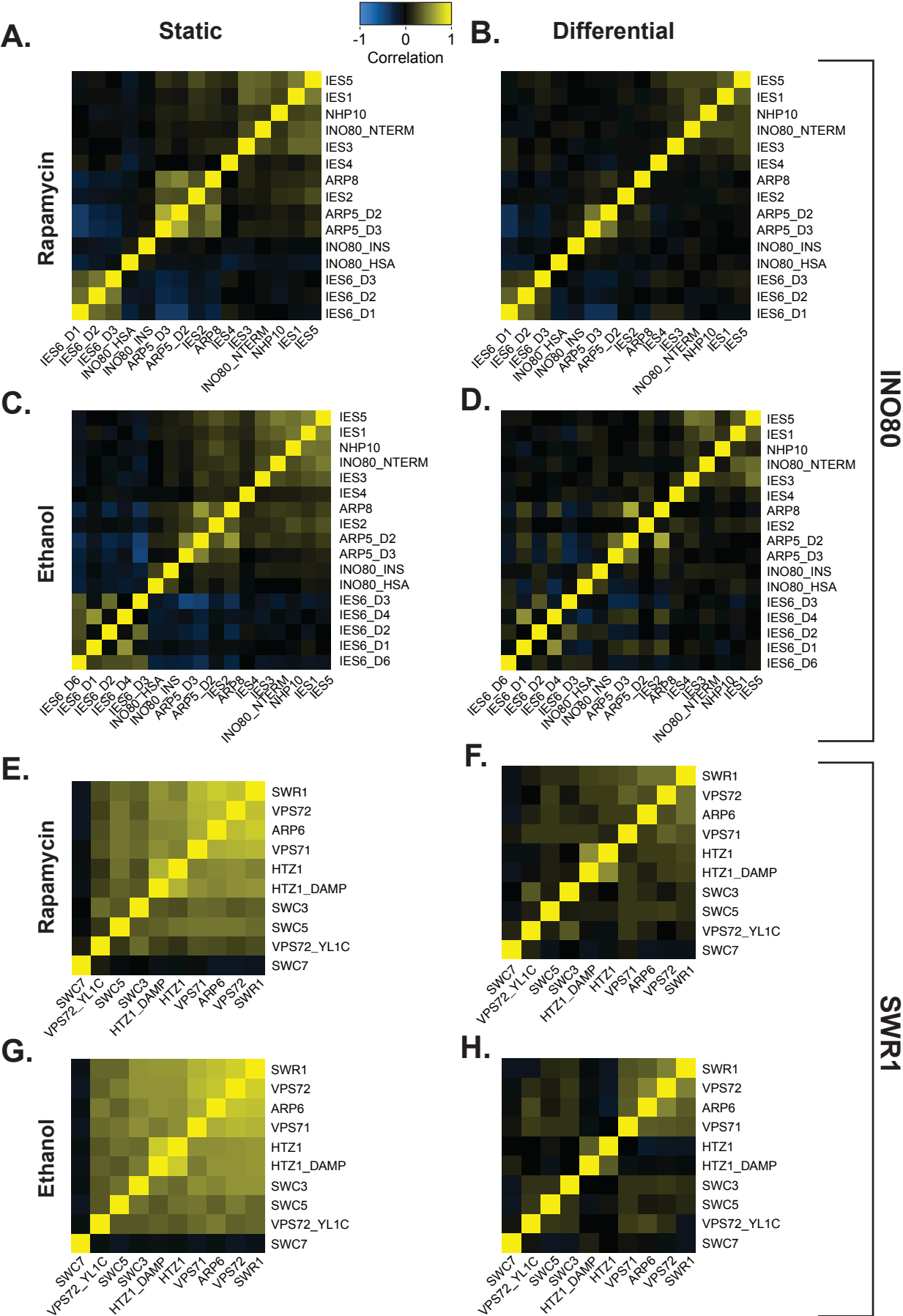

Supplement: S6 Fig — Heatmap illustrating pairwise Pearson correlations between INO80 (A-D) and SWR1 (E-H) complex subunit query strains across the test library, as in Fig 2A and 2B. Rapamycin static correlations (A and E) and differential correlations (B and F) are shown. Ethanol static correlations (C and G) and differential correlations (D and E) are shown. Strains are ordered as shown in Fig 2A and 2B and determined by untreated hierarchical clustering. (PDF) [file pgen.1007216.s006.pdf]

S7 Fig

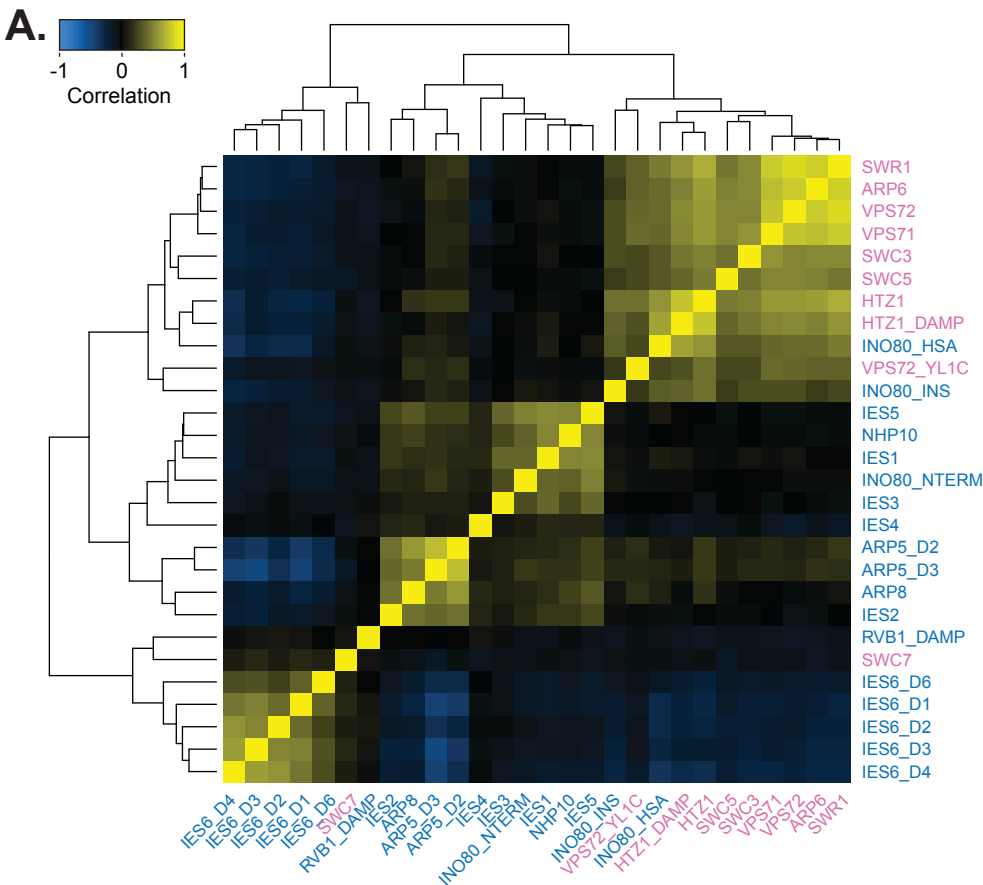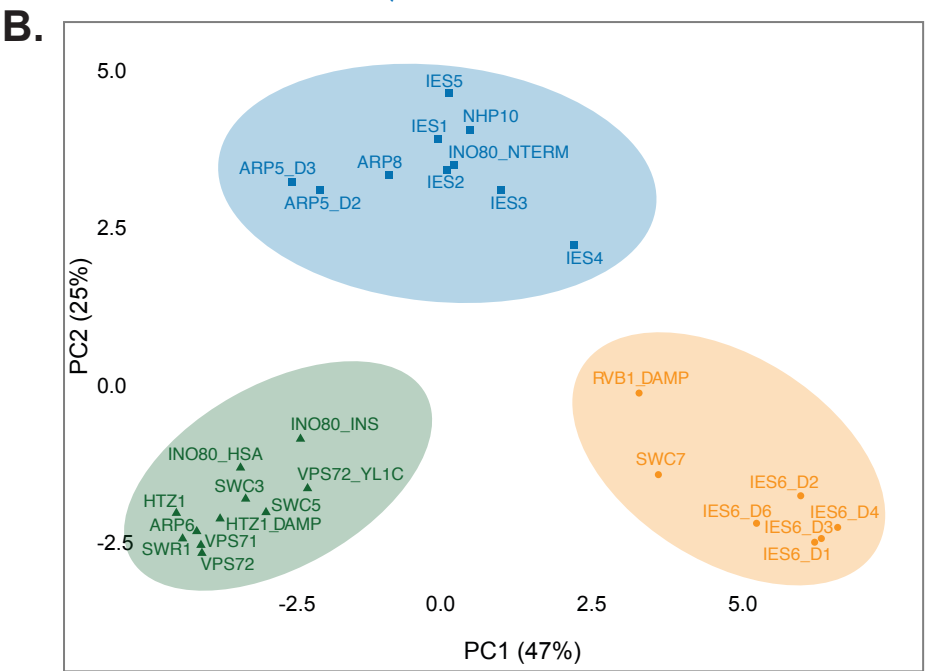

Supplement: S7 Fig — (A) Heatmap of Pearson correlation of INO80 and SWR1 complex subunit query strains in the untreated static condition, as in Fig 2A; colors delineate complexes as in Fig 1B. Mutants are complete deletions or domain deletions where indicated: INO80 N-terminal (NTERM), insertion (INS), and HSA deletions; ARP5 domain 2 and 3 (D2 and D3) deletions; and IES6 domain 1, 2, 3, 4, and 6 (D1, D2, D3, D4, D6) deletions. Decreased abundance by mRNA perturbation (DAmP) alleles are as described in [36]. Boxes outline subunit clusters identified by hierarchical clustering. (B) Principal component analysis (PCA) of INO80 and SWR1 complex subunit query strain Pearson correlations, as in Fig 2B. Colors indicate clusters identified by k-means clustering (k = 3). (PDF) [file pgen.1007216.s007.pdf]

S8 Fig

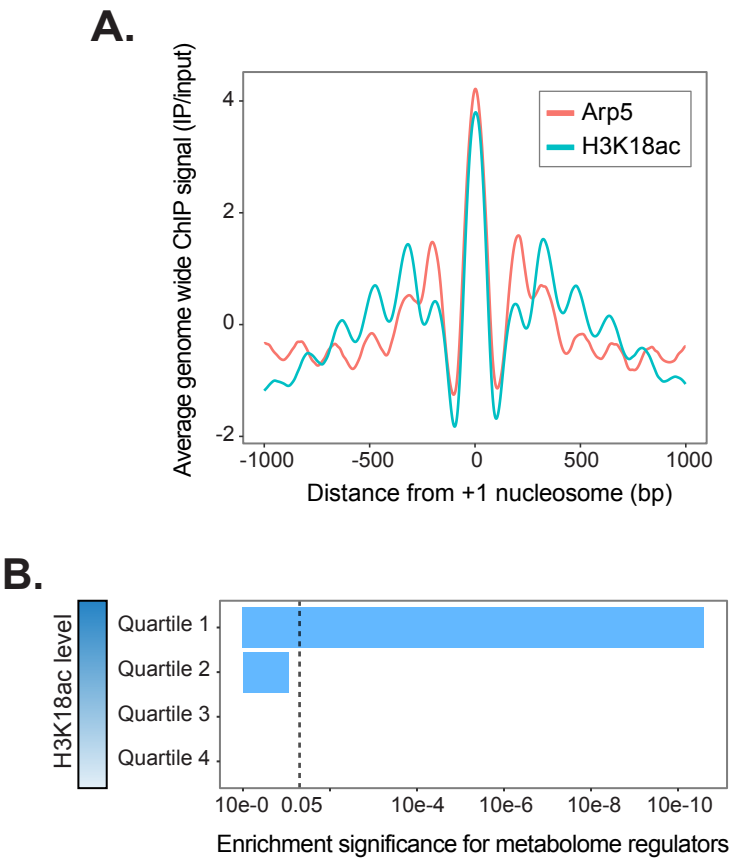

Supplement: S8 Fig — (A) Genome-wide average uniformly processed (see Materials and Methods) ChIP-seq levels ±1000 bp from +1 nucleosomes [76] of Arp5 [7] and H3K18ac [69]. (B) Genes with high H3K18ac levels at +1 nucleosomes are significantly enriched for regulators of the metabolome; significance was determined using a hypergeometric test. (PDF) [file pgen.1007216.s008.pdf]

S9 Fig

A.

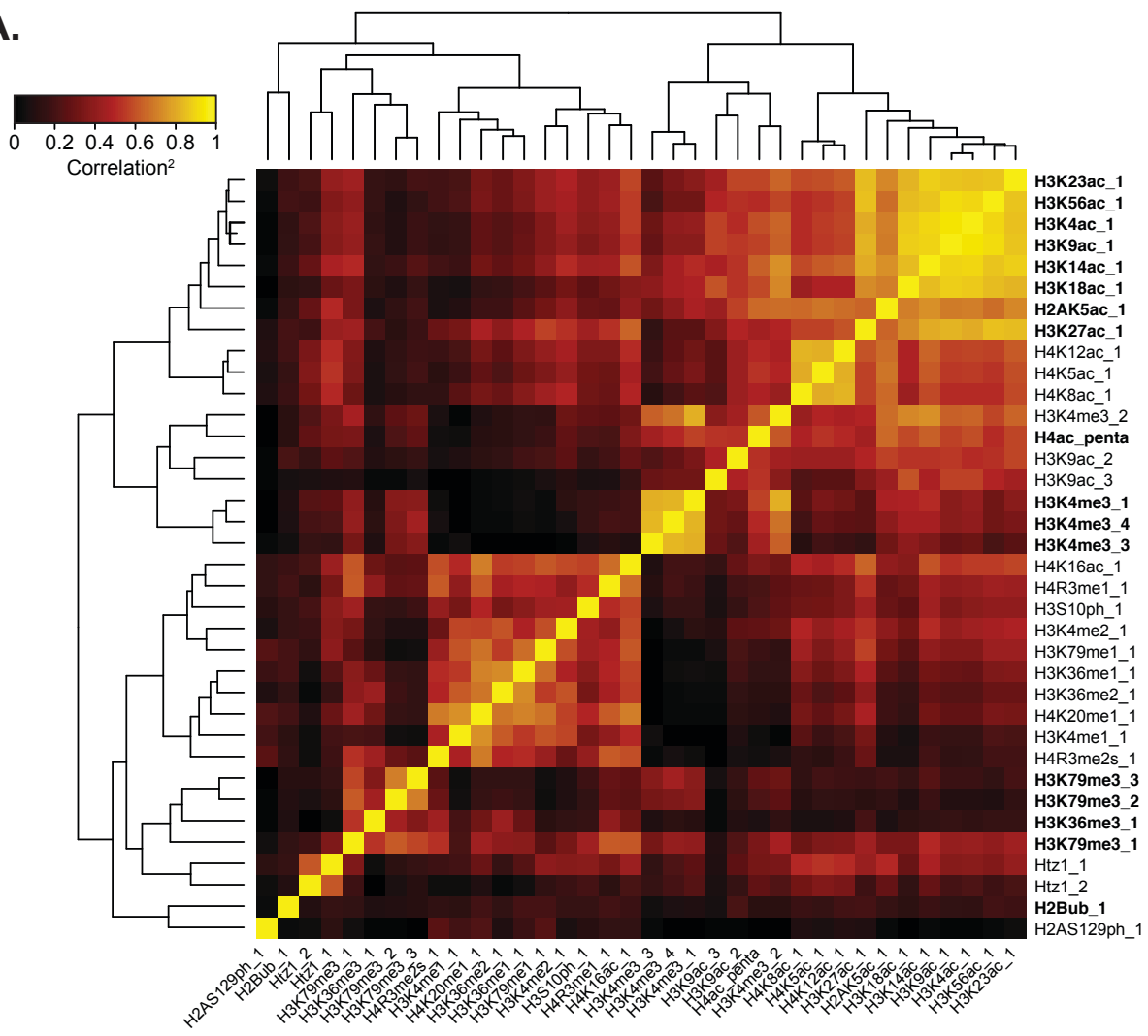

B.

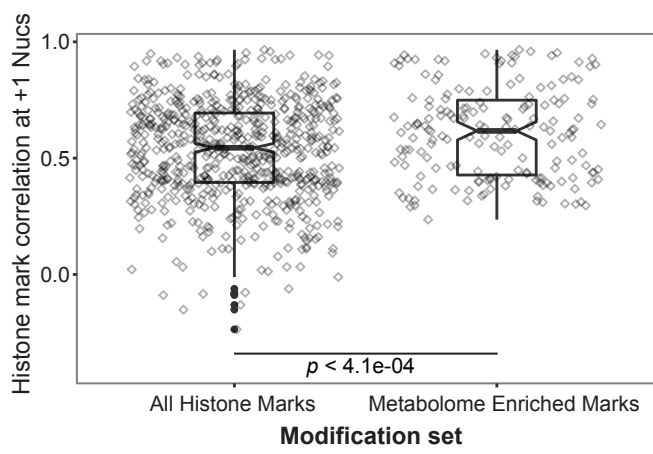

Supplement: S9 Fig — (A) Heatmap of pairwise squared Pearson correlations at +1 nucleosomes using uniformly processed published ChIP-seq data (see Materials and Methods). Modifications that have significantly high levels at the +1 nucleosomes of metabolome regulators and are enriched for metabolome regulators in their top quartile of +1 nucleosome levels are bolded. (B) Box and jittered scatter plots of correlations between all histone marks shown and the metabolome enriched marks bolded in (A). Significance is determined using a Wilcoxon rank sum test (p < 4.1e-4) and by Monte Carlo randomization test (p = 0.0419). (PDF) [file pgen.1007216.s009.pdf]

S10 Fig

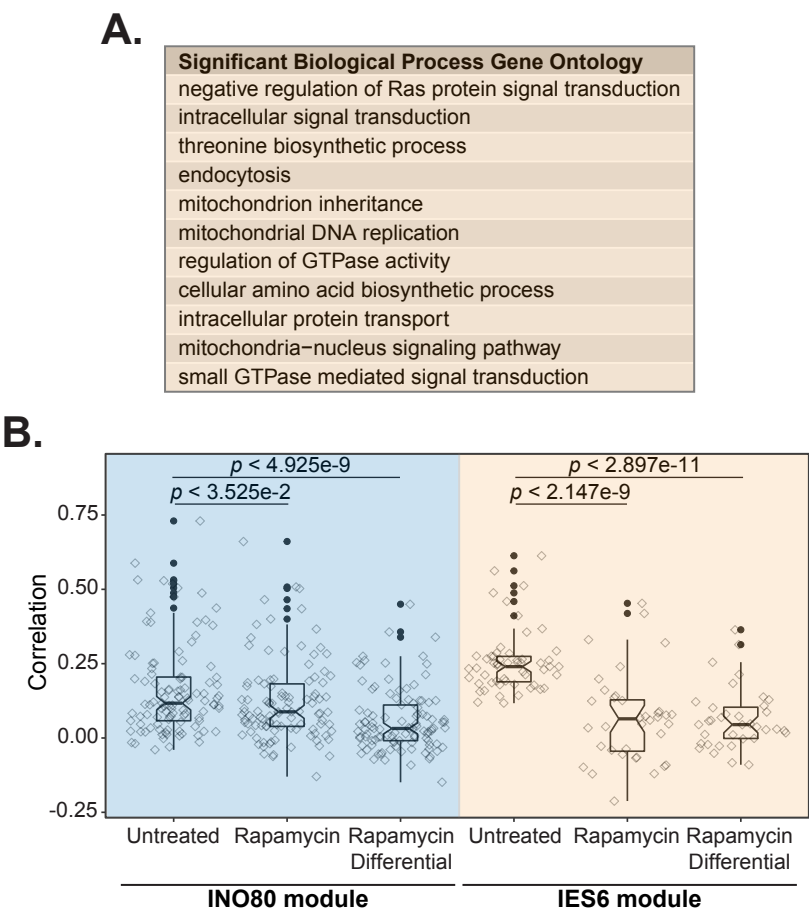

Supplement: S10 Fig — (A) Table showing select gene ontology (GO) terms enriched in test strains that significantly interact with the IES6 cluster query genes (FDR-adjusted hypergeometric test, p < .05). The complete list of significant GO terms is found in S7 Table. (B) Box and jittered scatter plots of correlations between query genes in the INO80 and IES6 expanded modules, shown in Fig 4A, in the untreated static, rapamycin static and differential conditions. Significance is determined using a Wilcoxon rank sum test. (PDF) [file pgen.1007216.s010.pdf]

**S11 Fig**

### INO80 - TOR pathway: Rapamycin Differential

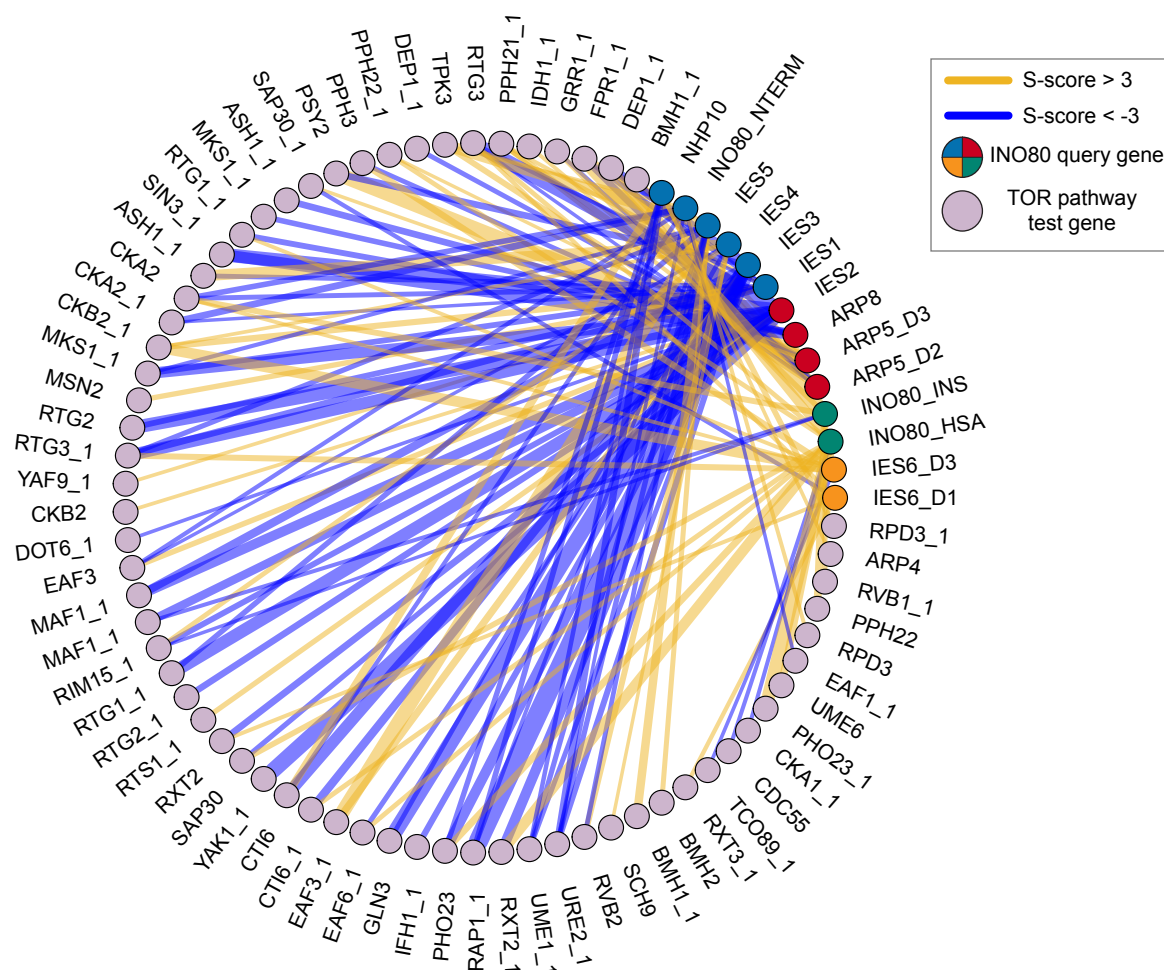

Supplement: S11 Fig — Genetic interaction network between INO80 subunit query strains and significantly interacting TOR pathway test strains in the rapamycin differential condition. Line width indicates strength of S-score, INO80 queries are colored according to modules identified in Fig 2. Network density is significantly high, p-value = 1.6e-4 by Monte Carlo randomization test. (PDF) [file pgen.1007216.s011.pdf]

S12 Fig

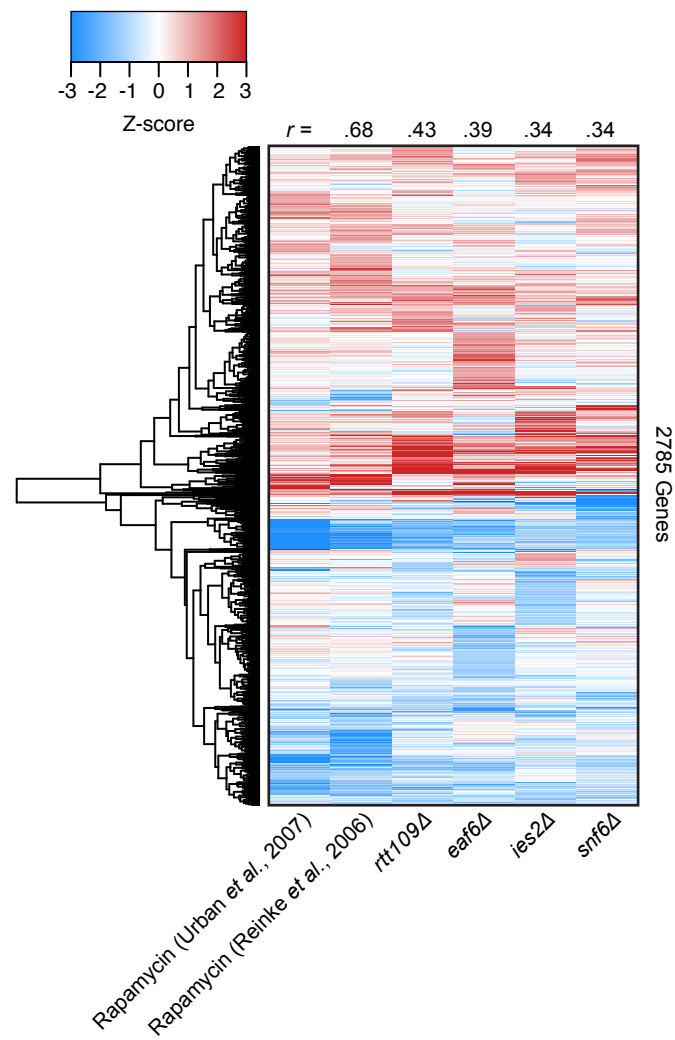

Supplement: S12 Fig — Log-transformed Z-scores of expression fold-change between untreated and treated wild-type cells or indicated deletion strains representing the top 5 highest correlating samples to rapamycin treatment from Urban et al. 2007 [54,59,104]. Genes with at least a Z-score of ±1.0 are plotted. Pearson correlations are shown for all genes (>5800) regardless of fold-change difference. (PDF) [file pgen.1007216.s012.pdf]
